# Challenges and Prospects of Plant-Derived Oral Vaccines against Hepatitis B and C Viruses

**DOI:** 10.3390/plants10102037

**Published:** 2021-09-28

**Authors:** Ana-Maria Madalina Pantazica, Lia-Maria Cucos, Crina Stavaru, Jihong-Liu Clarke, Norica Branza-Nichita

**Affiliations:** 1 Department of Viral Glycoproteins, Institute of Biochemistry of the Romanian Academy, 011254 Bucharest, Romania; ana.pantazica@biochim.ro (A.-M.M.P.); lia.cucos@biochim.ro (L.-M.C.); 2 Laboratory of Immunology, “Cantacuzino” Medico-Military National Research Institute, 011254 Bucharest, Romania; 3 Division of Biotechnology and Plant Health, NIBIO—Norwegian Institute for Bioeconomy Research, NO-1432 Ås, Norway

**Keywords:** HBV, HCV, oral vaccines, edible plants, mucosal immunity

## Abstract

Hepatitis B and C viruses chronically affect approximately 3.5% of the global population, causing more than 800,000 deaths yearly due to severe liver pathogenesis. Current HBV vaccines have significantly contributed to the reduction of chronic HBV infections, supporting the notion that virus eradication is a feasible public health objective in the near future. In contrast to HBV, a prophylactic vaccine against HCV infection is not available yet; however, intense research efforts within the last decade have significantly advanced the field and several vaccine candidates are shortlisted for clinical trials. A successful vaccine against an infectious disease of global importance must not only be efficient and safe, but also easy to produce, distribute, administer, and economically affordable to ensure appropriate coverage. Some of these requirements could be fulfilled by oral vaccines that could complement traditional immunization strategies. In this review, we discuss the potential of edible plant-based oral vaccines in assisting the worldwide fight against hepatitis B and C infections. We highlight the latest research efforts to reveal the potential of oral vaccines, discuss novel antigen designs and delivery strategies, as well as the limitations and controversies of oral administration that remain to be addressed to make this approach successful.

## 1. Introduction

Undoubtedly, vaccination is the primary and most effective tool in combating infectious diseases in both humans and animals; however, the success of immunization critically depends on achieving a broad level of coverage, sufficient to interrupt transmission of pathogens [1]. To reach this goal, a vaccine must not only be safe and efficient against the pathogen but also easy to manufacture, distribute (e.g., thermally stable) and administer (e.g., orally), as well as affordable to low-income populations. The fast advancement in COVID-19 vaccine development followed by a rapid global vaccination against the coronavirus (CoV-2) are good examples that demonstrate the importance of effective and safe vaccines for human and animal health. 

The majority of pathogenic microorganisms or antigens penetrate the body through the enormous mucosal surfaces which form the boundary between the interior of the body and the external environment [2]. However, most currently licensed vaccines are administered through systemic routes, by using intramuscular or subcutaneous injections, which trigger a protective systemic immune response but are less effective in eliciting antigen-specific immune responses at the mucosal surface [3]. In contrast, oral delivery of vaccines has the potential to enhance the mucosal immune response at the site of infection but can also trigger systemic immunity. In addition, oral immunization is noninvasive, safe, and simple, thereby ensuring better patient compliance and clinical practicality. These features promote oral immunization as a viable approach in controlling infectious diseases of global significance. However, the potency of oral vaccines against blood-borne pathogens that invade the host and disseminate by systemic routes is still a matter of debate.

In this review, we provide a concise update on the development of plant-based oral vaccines against two major human pathogens, the hepatitis B and C viruses, currently affecting more than 325 million chronic carriers worldwide [4]. We highlight the latest research efforts to unfold the full potential of mucosal vaccines in fighting these infections, discuss novel antigen designs and delivery strategies, as well as the weaknesses and controversies of oral administration that still remain to be addressed to make this immunization strategy successful. 

## 2. Mechanisms of Mucosal Immunity and Relevance for Fighting Systemic Viral Infections

As mentioned above, oral administration of vaccines offers several key advantages over parenteral immunization. It is therefore intriguing that only a few oral vaccines, mostly based on inactivated or live-attenuated pathogens, have made their way to clinical approval for use in humans [5]. 

One possible reason lies in the complexity of mechanisms regulating the mucosal immune system that have evolved to achieve and maintain tolerance against self-antigens and a multitude of foreign antigens from microflora and food [6]. While this physiological process protects the organism from damaging inflammatory responses in the intestine, it represents a major impediment for the development of oral vaccines. 

At the intestinal mucosa, adaptive immune responses to antigens are initiated in gut-associated lymphoid tissues (GALT), which include the Peyer’s patches (PPs), cecal patches, colonic patches, isolated lymphoid follicles (ILFs), cryptopatches, and draining mesenteric lymph nodes (MLNs) [7].

The mucosal epithelial surface has a unique structure, containing microfold (M) cells specialized in antigen capturing and transport, overlying organized mucosal lymphoid tissue, lymphocytes, plasma cells, dendritic cells (DCs), and macrophages. Moreover, mucosal epithelial cells express various molecules with different functions: (a) polymeric immunoglobulin receptor (pIgR), which binds dimeric IgA (dIgA); (b) polymeric IgM, which mediates the transport of soluble immune complexes to the apical surface after endopeptidase cleavage; (c) major histocompatibility complex (MHC) class I and II molecules responsible for antigen presentation; and (d) a variety of immune response regulators such as cytokines, chemokines, and adhesion molecules [8,9,10,11].

**Figure 1:**
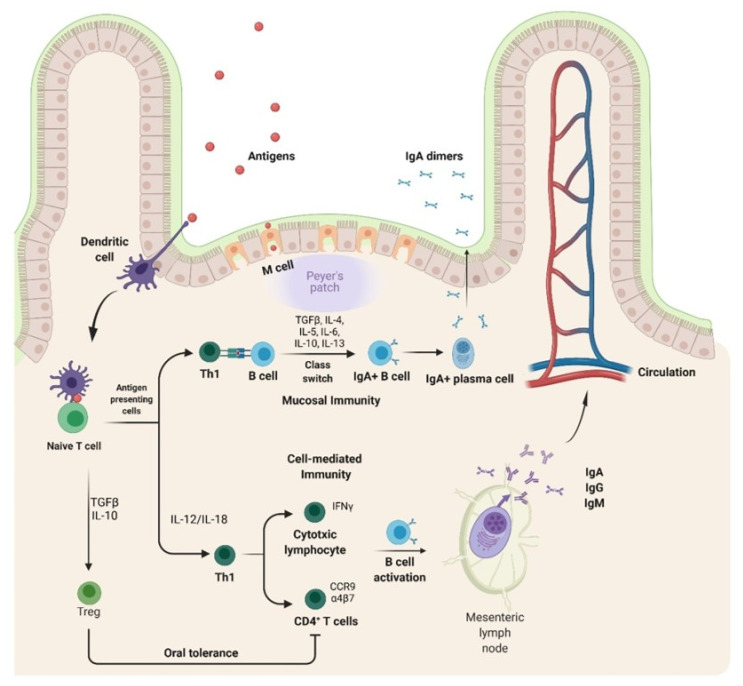
Mechanism of mucosal immunity.

Upon ingestion, antigens face the first critical barrier on the way to GALT, having to get through the low gastric pH and the protease rich environment of the gastrointestinal tract. Surviving antigens are taken up from the intestinal lumen by M cells and transported to the underlying DCs, the professional antigen presenting cells in PPs (Figure 1).

The efficacy of oral vaccines is influenced by the antigen delivery and the immunization protocols which influence activation of naive CD4+T lymphocytes. Orally-administered HBV/HCV antigens can be either endocytosed by specialized M cells overlying the organized mucosal lymphoid tissue or specifically captured by DCs through the epithelial cell layer, via dendrites projection into the lumen. Regardless, the interaction between DCs and antigens triggers the priming of naïve T cells. Primed helper T cells then interact with antigen specific B cells, inducing class-switching to immunoglobulin secreting cells. Notably, the isotype switching of B cells to surface IgA positive (Bα) B cells and then differentiation to polymeric IgA producing plasma cells require the presence of TGFβ. Moreover, the presence of additional cytokines, such as IL-4, IL-5, IL-6, IL-10, and IL-13, as well as various receptors and ligands of innate and acquired immunity, are required to support the immune response induced by orally-administered vaccines. For cell-mediated immunity, activated T cells can become tissue-resident memory T cells or circulating-memory T cells. The differentiation and regulation of the cellular immune response is driven by cytokines such as IL-12/IL-18, which are produced by macrophages and DCs and promote the Th1 cellular immune response and IFNγ production. Moreover, CD4^+^ T cells which become antigen responsive express the homing markers α4β7 integrin and CCR9 chemokine receptors and can migrate to the mucosa. Activated B cells then enter systemic circulation and migrate to distant effector sites where they differentiate and mature into plasma cells. Finally, mucosal DCs can induce Tregs via production of TGF-β and IL-10, thereby mediating immune tolerance via suppression of Th1 and Th2 responses (figure created with BioRender.com accessed on 9 September 2021).

Processed antigens are then loaded onto the MHC and presented by migratory DCs that travel to the inductive sites of PPs or MLNs, thereby providing costimulatory signals to activate naive CD4+ T cells. These cells become antigen responsive once they express α4β7 integrin and CCR9 chemokine receptors with homing affinity to the mucosa, the effector site. Primed helper T cells further interact with antigen-specific B-cells that undergo class-switching to become immunoglobulin-secreting cells. The isotype switching of B cells to surface IgA (sIgA+) and differentiation to polymeric IgA-producing plasma cells require the presence of transforming growth factor β (TGFβ). Importantly, depending on the antigen type, a cellular immune response can also be triggered by activated T cells which may become tissue-resident or circulating-memory T cells, thus controlling the infection via the production of a specific set of cytokines and recruitment of other immune cells. Differentiation and regulation of the cellular immune response are driven by cytokines such as IL-12/IL-18 which are produced by macrophages and DCs that, in turn, promote Th1 cellular immune response and IFNγ production. In contrast, a Th2 response is characterized by the production of a different set of cytokines such as IL-4, IL-5, IL-6, IL-10, and IL-13, which further support humoral immunity.

This complex signaling leading to lymphocyte activation may occur in any individual PPs or MLNs, but, depending on the context of antigenic exposure can either trigger antigen-specific humoral and cellular immune responses or be oriented to immune tolerance, another major drawback of oral vaccination [12,13] (Figure 1). T regulatory cells (Tregs) are the major suppressors of effector T-cell differentiation and proliferation by producing IL-10 and TGF-β, thus maintaining the intestinal homeostasis through promoting immune tolerance to commensal microbiota and food antigens [14]. Oral tolerance is currently tackled by ensuring sufficient protection of antigens against the gastrointestinal environment and microbiota interactions, high antigen loading or encapsulating capacity of vehicles, prolonged exposure of antigens to antigen-presenting cells, sufficient targeting ability to intestinal cells, especially M cells, and enough production of long-term mucosal and systemic effect, while maintaining adequate safety [15,16].

## 3. Oral Vaccines Produced in Edible Plants, Advantages, Disadvantages and Lessons Learnt

Plants as an ideal green factory have been exploited in the past three decades for the production of various biopharmaceuticals and vaccines for human and animal health as well as high value proteins [17,18,19,20,21]. 

Compared to traditional platforms for recombinant protein expression (i.e., bacterial, mammalian, or insect cells), plants offer several key advantages. As eukaryotic organisms, plants enable posttranslational modifications of proteins. With regard to vaccine production, plant-specific N- and O-glycosylation have various effects on recombinant antigens, increasing or attenuating their immunogenicity as compared to their mammalian cell counterparts [22,23]. Processing of complex transmembrane viral proteins, assembly of functional oligomers, and virus-like particles (VLPs) are also possible in plants [21,23]. Plants’ components associated with VLPs may have adjuvant properties increasing the protein immunogenicity and thus preventing the use of additional adjuvants. Among the disadvantages of plants as vaccine production platforms are the requirement for complex (Good Manufactory Practice-GMP) infrastructure for downstream processing of plant extracts, the low production and often unpredictable protein yield, and the limited regulatory framework. A comprehensive review of the advantages and disadvantages of plant made vaccines has been very recently published, during the revision of this manuscript [24]. 

Edible plants are of special importance for the production of antigens [18,25] and have been long exploited for the production of vaccines for humans and animals through oral administration [17,24,26]. To date, lettuce-based platforms for drug and vaccine production are well established and are being used for the successful production of several candidate drugs and vaccines [17,18,27,28]. Among the plant genetic engineering technologies developed, plant transient expression, in particular the production of VLPs and stable chloroplast genome engineering, are fully established for lettuce-based edible plant production platforms. Drugs and vaccines for oral administration produced in lettuce have several advantages when compared with the same products made in other plant species, e.g., tobacco, *Nicotiana tabacum* or *N. benthamiana* plants, which need to be subsequently purified before being tested. Previous studies and techno-economic analyses have revealed that the cost for downstream processing of plant derived medicines and vaccines accounts for almost 80% of the total vaccine production cost [17,29]. Given that low and middle income countries (LMICs) are often devastated by infectious diseases such as the COVID-19 pandemic, affordable vaccines are essential to control infections and save lives. Oral vaccines made in edible crops do offer this economic advantage. Stability at room temperature with no requirement for cold storage and easy transportation are also worth mentioning as unique advantages of oral vaccines made in edible-plants. These are of particular significance for LMICs where cold storage and vaccine transport can be rather limited. In addition, the employment of recently developed freeze-drying [30] or lipid depletion technologies [31] have also significantly improved the shelf-life of these vaccines, eliminating the need for cold-storage, facilitating distribution and further improving their availability. An encouraging case was reported recently demonstrating the first protein drug encapsulated in plant cells approved by the FDA demonstrating the advantages and potential of oral vaccines made in edible crops [17]. Another significant advantage of oral vaccination is the simplification of the immunization protocols, normally requiring multiple injectable doses to ensure sufficient protection, as it is the case for the HBV vaccine. An oral vaccine used as a booster (boost vaccine) after priming with an injectable vaccine was reported [21,25]. 

Despite the advantages and potential of oral vaccines produced in edible plants, there are several challenges and hurdles that must be dealt with and more clinical trials of plant-made oral vaccines are required. One of the challenges is the efficiency of the immune response of a vaccine administrated through an oral route. The second challenge is the stability of oral vaccines when passing through the gastrointestinal tract. Van Eerde et al. performed an in vitro gastrointestinal digestion analysis and revealed that the lettuce-made dengue EDIII-1-4 antigens are well protected when passing through the oral and gastric digestion phases but underwent degradation during the intestinal phase [20]. The targeted release of oral vaccine antigens into the human intestine and vaccine protection efficacy are therefore among the other challenges encountered in edible vaccine development [25]. 

Another significant disadvantage regarding the production of oral vaccines is the yield of recombinant antigens, which varies considerably depending on the characteristics of the candidate antigen, the nature and biomass of the edible plant host, protein stability, and the technology utilized (i.e., transient expression or stable chloroplast transformation). Establishing optimal dosages and effective prime-boosting immunization schemes to induce sufficient immune response and prevent immune tolerance are also major issues that need to be addressed. Current approaches considering improvement of adjuvants and antigens delivery to the intestinal immune cells, together with recent technological advancements in plant genetic engineering, increasing expression of recombinant proteins in plant cells [32], might overcome some of these challenges in the future. 

Finally, the burden of the regulatory framework associated with the use of genetically modified plants for the development of oral vaccines needs to be evaluated. Biosafety regulations and guidelines of molecular farming for production of biopharmaceuticals need to be made applicable, while new legal directives must regulate a smooth transition of plant-derived oral vaccines research to clinical trials and marketing. Therefore, international efforts in conducting more clinical studies and sharing the data and outcomes from the clinical trials of oral vaccines are of importance for the future development of oral plant vaccines against viral infections such as HBV and HCV [18,33]. 

## 4. Features of HBV and HCV Biology Relevant for Vaccine Development: Viral Antigens and Vaccine Candidates

Approximately 3.5% of the world population is chronically infected with either HBV or HCV and over 800,000 people die yearly due to liver complications such as cirrhosis and hepatocellular carcinoma, flagging these pathogens as leading human health threats [4,34,35]. Understanding the structure and mechanisms employed by these viruses to target the hepatocyte, their replication host, are essential for the development of preventive vaccines.

### 4.1. HBV 

HBV is an enveloped DNA virus, a member of the Hepadnaviridae family, and was first described in 1970 by Dane et al. as a 42 nm in diameter particle [36]. Its tiny genome consists of a partially double-stranded relaxed circular DNA of 3.2 kb, containing four partially overlapping open reading frames (ORFs), namely the core (pre-C/C), envelope (pre-S/S), polymerase (P), and transactivating protein X (X) genes, altogether encoding seven viral proteins [37,38].

The Pre-S/S ORF encodes the overlapping large (L), middle (M), and small (S) envelope glycoproteins, collectively called HBV surface antigens (HBsAg). These proteins share the common S-domain displaying the major antigenic determinant that triggers production of virus-neutralizing antibodies [39]. The S protein is the most abundant constituent of the viral envelope, but also self-assembles into subviral particles (SVPs) lacking DNA, which are produced in great excess over mature virions. The M and L proteins contain additional domains at their N-terminal end, the preS2 and preS1, respectively [40]. Notably, a 48 amino acids region in the N-terminal region of the pre-S1 domain mediates HBV attachment to its receptor, NTCP, and is a major target for virus neutralization antibodies [41,42,43]. 

SVPs are noninfectious but highly immunogenic due to their particulate nature, two features promoting these particles as ideal candidates for vaccine development. Indeed, the first HBV vaccine licensed in 1982 contained SVPs purified from inactivated plasma of HBV carriers [44]. This was later replaced by a second generation of HBV vaccines using recombinant S-HBsAg produced in yeast [45]. These vaccines have been commercially available since 1986 and have significantly contributed to the reduction of chronic HBV infection worldwide [46]. However, approximately 10% of the vaccinated population fails to develop appropriate levels of protective antibodies due to either genetic resistance, older age at immunization, severe liver and renal disease, immunosuppression, or simply lack of compliance to the complex 3-dose administration schedule [47]. In addition, the duration of the immune response triggered by current HBV vaccines is questioned by recent epidemiologic data indicating that protective immunity might be lost in some cases at 15 years post-primary vaccination [48]. 

To address these issues, a new generation of HBV vaccines including the L and M proteins in addition to S have been produced and are currently awaiting FDA approval. Clinical trials have concluded on the ability of these novel vaccines to induce higher antibody titers with a larger spectrum of antibodies as compared with traditional S-HBsAg-based vaccines [49]. However, these antigens are not easily expressed in bacteria or yeast, thereby requiring costly production in mammalian cells, which may hamper large-scale administration. A currently investigated alternative takes advantage of the structural properties of the S protein and employs it as a scaffold to expose relevant preS1/preS2 epitopes in chimeric SVPs produced in various expression systems [23,50,51,52,53]. 

The complexity of the vaccine research field and industry is illustrated by the recent success of the only FDA-approved HBV vaccine in more than two decades since the first commercial formulation. This novel vaccine, available since 2018, still contains yeast-derived HBsAg, but in combination with cytosine phosphoguanosine (CpG) 1018, a more immunogenic adjuvant [54]. Notably, the protocol involves administration of two injections over the course of one month, which is a great improvement of the three doses over six months classical HBV vaccine. This can potentially increase patient compliance and therefore the number of people who are protected against HBV; however, it can only be administered to individuals over the age of 18 [55].

### 4.2. HCV

HCV, an enveloped, single stranded positive RNA virus and the main representative of the Flaviviridae family, was discovered in 1989 [56]. The 9.6 kb HCV genome consists of a long open reading frame encoding a polyprotein of about 3000 amino acids which is processed by cellular and viral proteases into 10 structural (core, E1, E2) and non-structural proteins (p7, NS2, NS3, NS4A, NS4B, NS5A, and NS5B) [57]. The viral envelope contains the complex type 1 transmembrane glycoproteins E1 and E2 that form a heterodimer stabilized by disulfide bonds.

HCV entry is a complicated process involving sequential interactions of the viral particles with several host cell receptors. Infection is initiated by virus concentration at the basolateral membrane of the hepatocyte following binding of the viral particle associated lipoproteins to LDL receptors [58,59]. Exposure of viral envelope glycoproteins at the virus surface then allows for a specific interaction with SR-BI, CD81, and CLDN1 forming the co-receptor complex on the hepatocyte plasma membrane, which facilitates virus internalization through clathrin-mediated endocytosis [60,61]. 

As a prerequisite for infection, the HCV entry process is an excellent target for a protective immune response. It is therefore not surprising that most antibodies with broad virus neutralizing activities to different HCV genotypes recognize conformational epitopes in E2 or the E1E2 heterodimer, making these antigens the primary candidates for vaccine development [62].

Despite tremendous research efforts towards the development of a prophylactic treatment, a vaccine against HCV is not currently available. There are several reasons that could explain the failure of this scientific endeavor hitherto.

One main challenge is the immense HCV genetic diversity, with seven known genotypes and more than 80 subtypes [63], exceeding even the diversity of the human immunodeficiency virus-1. Moreover, host immune pressure generates a diverse quasispecies of related but distinct variants at the nucleotide level, within each infected individual [64,65,66]. This explains why even though spontaneous HCV infection clearance occurs in 25% of infected individuals [67], recurrent infections following new exposures can happen. Thus, a primary cleared infection does not ensure a full protective immunity [68,69]. 

Despite the vast heterogeneity of the HCV genome, vaccination remains feasible because viral replication can trigger specific HCV T-cell and humoral responses at the beginning of the infection [70]. To be effective against a pathogen with such sequence variability, the ideal vaccine should induce broad immune responses that would target highly conserved regions of the antigenic determinants. However, the role of antibodies in neutralizing HCV appears much more complex. Probably, a more realistic approach towards developing an HCV vaccine would be to consider its potential use as a chronic infection suppressor or immunological booster.

Another challenge in developing an HCV vaccine resides in its ability to evade the host’s adaptative immune response using different strategies. The first is the use of the hypervariable region 1 (HVR1) of E2, which elicits non-neutralizing antibodies acting as an immunodominant epitope. It is also believed that HVR1 shields more conserved epitopes of E2 that are critical to CD81 binding [71,72]. Epitope shielding is also achieved through heavy glycosylation of E2 which contains 11 N-linked glycans [73]. Glycan shifting also appears in the context of antibody escape mutants which indicates an important role in the HCV defense mechanisms [74,75]. 

As the target of nAb response is represented by envelope proteins, full length or soluble versions of the recombinant (r) E1E2 dimer or E2 alone have been the major vaccine candidates [76]. The low level of expression of this antigen in different expression hosts, achieving the correct folding and processing were the main challenges of these strategies. Preclinical tests have indicated that immunization of chimpanzees with rE1E2 triggered humoral response along with protective immunity [77]. This study moved to human trials and revealed that rE1E2 was safe in humans and elicited an immune response, but disappointingly, not a neutralizing one [78]. More recently, it has been shown that E1E2 can be expressed in their native assembly state through the insertion of a scaffold instead of the transmembrane domain that enforces assembly while aiding in expression and purification [79]. Furthermore, this antigen induced a virus neutralizing immune response, promoting this strategy as a promising alternative for future E1E2 vaccine designs.

## 5. Could Oral Vaccines Have a Future in Controlling HBV/HCV Infections?

As discussed above, eliminating parenteral administration or reducing the number of injections from the vaccination schedule would certainly mark a great progress towards increasing vaccine acceptance and coverage. Despite the controversies regarding their efficacy and the challenging regulatory barriers, research in edible plant-derived vaccines indicates their potential to overcome some of the difficulties associated with conventional vaccines. 

### 5.1. Oral Vaccine Candidates against HBV

Given the complexity of the HBsAg folding, the delivery of the first proof-of-concept demonstrating expression of this antigen in plants (i.e., *Nicotiana tobaccum*) was considered a real breakthrough in molecular farming [80]. Moreover, the plant-expressed HBsAg was able to elicit a similar immune response to that obtained for the yeast-derived counterpart, when administered intramuscularly in mice [81]. This success has attracted a tremendous interest towards employing the plants as antigen factories and has led to further research investigating other plant species as hosts for viral antigen production, as well as the potential use of plant-derived antigens for oral immunizations. Notably, while S-HBsAg production has been investigated in many plant species, the production of M- and L-HBsAg in plants has been comparably less intensively researched, possibly due to their cellular toxicity when expressed alone. In recent years, the production of the HBV core antigen (HBcAg), which is also known to elicit a strong immune response and protective antibodies when produced in plants, has also received increasing interest.

Of the vast body of research in this field, this review focuses on the work conducted on edible plant-produced HBV antigens that provides data on the immunogenic properties in tissue cultures, animal models, or clinical studies (summarized in Table 1).

The first study which evaluated the ability of plant-produced HBsAg to function as oral vaccines was published in 1999 by Kapusta et al. [82]. In this study, mice fed transgenic Lupin callus expressing HBsAg produced significant levels of HBsAg-specific antibodies, underlying the importance of antigen doses and immunization protocols. The HBV antigen was later produced in potato tubers [83,84,85,86,87], tomatoes [88,89], maize [31,90,91], and lettuce [30,33,82,92,93,94], and immunizations were performed in the presence of adjuvants, such as the cholera toxin (CT), which activates GALT enhancing delivery of the antigens to the immune system. However, currently there are no mucosal adjuvants approved for human use, which is a critical issue regarding the use of edible vaccines in humans. 

Interestingly, tomato-expressed HBsAg did not elicit an immune response in mice, when administered orally, but it could work as a booster vaccine [88]. However, this study was limited by low HBsAg expression levels and the amounts which could be consumed in 24 h. Another study evaluated tomatoes expressing M-HBsAg, which were shown to elicit significant antibody titers (<200 mUI/mL) that were maintained at 3-months post immunization [95].

**Table 1:** Oral HBV plant vaccines with proven immune responses.

Plant System	HBV Antigen	Adjuvant	Immunization Protocol	Characteristics of the Immune Response	Reference
Lupin (*Lupinus luteus*)	HBsAg	None	Oral administration in mice of two doses at 1-month intervals	IgG antibodies in serum (maximum 19 mIU/mL)	[82]
*Nicotiana benthamiana*	HBcAg	None	Oral administration in mice of 2 doses via oral gavage at 2-week intervals followed by boost via intranasal administration at week 12	IgG antibodies in serum and IgA antibodies in feces	[96]
Potato	S-HBsAg	Cholera Toxin	Oral administration in mice of three doses (1 dose/week)	IgG antibodies in serum (maximum 73 mIU/mL for primary response; maximum 1680 mIU/mL after boost injection with subimmunogenic yeast-derived rHBsAg)	[83]
		None	Oral administration of 2 (28-day interval) or 3 doses (14- day interval) in humans previously vaccinated with licensed HBV vaccine	IgG antibodies in the serum of 57.57% (19/33) of volunteers (maximum 4785 mIU/mL)	[97]
		Cholera Toxin	Oral administration in mice of 3 doses (1 dose/week) followed by intraperitoneal boost with subimmunogenic dose of yeast-derived rHBsAg after 5 weeks	IgG antibodies in serum (maximum 700 mIU/mL)	[84]
		Cholera Toxin	Oral administration in mice of 3 doses (1 dose/week)	IgG antibodies in serum (maximum 103 mIU/mL for primary response; maximum 3300 mIU/mL after boost injection with subimmunogenic yeast-derived rHBsAg)	[87]
		None	Oral administration in mice of 3 doses (1 dose/week) followed by intraperitoneal boost with subimmunogenic dose of yeast-derived rHBsAg after 8 weeks	IgG antibodies in serum (maximum 179 mIU/mL for primary response; maximum 350 mIU/mL after boost injection with subimmunogenic yeast-derived rHBsAg)	[86]
	M-HBsAg	Cholera Toxin	Oral administration in mice of 3 doses (1 dose/week) followed by intraperitoneal boost with subimmunogenic dose of yeast-derived rHBsAg after 5 weeks	IgG antibodies in serum (maximum 700 mIU/mL),	[84]
		Cholera Toxin	Oral administration in mice of 3 doses (1 dose/week) followed by intraperitoneal boost with subimmunogenic dose of yeast-derived rHBsAg after 8 weeks and *E. coli*-derived recombinant preS region after 32 weeks	IgG antibodies in serum (maximum 558 mIU/mL at week 21) and IgA antibodies in feces	[85]
Tomato	S-HBsAg	None	Oral administration in mice every day for 4 weeks followed by intraperitoneal boost with subimmunogenic dose of yeast-derived rHBsAg	No IgG antibodies in serum	[88]
			Oral administration in mice as a boost at 3–5 weeks after intraperitoneal boost with subimmunogenic dose of yeast-derived rHBsAg	IgG antibodies in serum increase after oral boost	
	M-HBsAg	None	Oral administration in mice of 2 doses at 2-week intervals.	IgG antibodies in serum (<200 mIU/mL) and IgA antibodies in feces	[95,98]
Lettuce (*Lactuca sativa*)	S-HBsAg	None	Oral administration in humans of 2 doses at 2-month intervals	IgG antibodies in serum >10 mIU/mL in 2/5 volunteers	[82]
		None	Oral administration in humans of 3 doses, the second at 1 week post-immunization and the third at 4 weeks post-secondary immunization	Maximum IgG antibodies in serum of 6.3 mIU/mL	[92]
		None	Oral administration in mice 30 or 60 days	IgG antibodies in serum (maximum 38 mIU/mL) and IgA antibodies in feces	[93]
		None	Oral administration in mice of PBS-suspended lyophilized tissue at 42 days after priming with subimmunogenic dose of commercial vaccine	IgG antibodies in serum (maximum 193 mIU/mL)	[30]
		None	Oral administration in mice of 2 doses at 4-week intervals	IgG antibodies in serum (maximum >3 mIU/mL)	[94]
		None or Cholera toxin B-subunit or saponins from quillaja bark or 10% *v/v* alhydrogel	Oral administration in mice of 2 doses at 6-week intervals after priming with subimmunogenic dose of commercial vaccine	IgG antibodies in serum (maximum >500 mIU/mL for lyophilizate and >800 mIU/mL for tissue extract)	
	S/preS1^21-47^-HBsAg	None	Oral administration in mice of 4 doses, 3 at 1-week intervals and the final on day 68	IgG antibodies in serum that were able to neutralize HBV infection in in vitro infection models	[33]
Maize	S-HBsAg	None or LT(R192G/L211A)	Oral administration in mice of 3 doses (0.83 mg/day for 3 consecutive days) at 2-week intervals at 13 weeks post-priming with subimmunogenic dose of commercial vaccine	IgG antibodies in serum (3003 mIU/mL without adjuvant or 4632 mIU/mL with adjuvant) and IgA antibodies in feces	[90]
		None	Oral administration in mice of 2 booster doses at day 112 and 126 post-priming with subimmunogenic dose of commercial vaccine	IgG antibodies in serum and IgA antibodies in feces	[31]
		None	Oral administration in mice of 4 booster doses at 13-, 15-, 47-, and 50-weeks post-priming with subimmunogenic dose of commercial vaccine	IgG antibodies in serum (maximum 12,755 mIU/mL) and IgA antibodies in feces	[91]

Maize is also an attractive expression system for oral vaccines, as it is more palatable than potatoes and ensures delivery of higher doses of antigen in a smaller amount of plant material. Oral administration of maize expressing HBsAg following immunization with a commercial HBV vaccine has led to a significant increase in antibody titers (>3000 mUI/mL), even in the absence of adjuvants [90]. High IgA titers were also found in feces, suggesting activation of the mucosal immune response, which may help protect parenteral non-responders. Notably, novel strategies to prolong the shelf-life of maize vaccines without affecting antigen properties, such as lipid extraction, have been developed [90].

Finally, *L. sativa* (lettuce) has proved of particular interest for the development of edible vaccines as it is generally consumed raw, thereby eliminating the issues regarding palatability and degradation of antigenic structure due to cooking. Additionally, freeze-drying techniques have been developed which permit the long-term storage and concentration of plants expressing HBsAg, without compromising the characteristics of SVPs [30]. Moreover, studies have shown that *L. sativa* may have intrinsic anti-HBV properties, due to its high glucosidase content [99]. Thus, oral administration of transgenic lettuce expressing HBsAg was shown to elicit both systemic and mucosal immune responses in mice [93] using a low-dose and an extended administration immunization protocol. This is of particular interest as studies have shown that high expression of HBV viral envelope proteins in *L. sativa* hinders their assembly into SVPs [100], possibly by overloading the ER folding pathway. Moreover, studies have also evaluated the effects of different immunization protocols using lyophilized *L. sativa* expressing HBsAg, mainly by varying the administered dose, delivering adjuvanted or non-adjuvanted plant tissue, and by using an oral immunization protocol vs. oral boost post-priming with a commercial vaccine [94]. The results showed that lower doses induced a more robust immune response and that oral administration of *L. sativa*-expressed HBsAg is most effective as a boost after priming with a subimmunogenic dose of the commercial vaccine. 

During the past several years, we have exploited a lettuce-transient expression platform for the production of oral HBV and HCV vaccines followed by immunological studies in mice [18,20,23]. Our results indicated that oral administration of unadjuvanted lettuce containing chimeric S/preS121-47 SVPs induced significant serum antibody titers which inhibited HBV infection in a cell culture infection model [33]. However, mucosal responses were not detected in our study, suggesting that further optimization of immunization protocols is necessary. 

Transgenic *L. sativa* was also used in one of the first studies evaluating the immunogenicity of edible vaccines in humans [82], in which three human volunteers were fed two doses of lettuce containing HBsAg. They found that two of the three volunteers developed antibody titers of >10 mUI/mL, which is considered to be protective. However, antibody levels dropped significantly at 4-weeks post-immunization. The ability of *L. sativa*-expressing HBsAg to induce an immune response in humans was further investigated by a later study, involving 12 participants who were fed transgenic *L. sativa* in three doses. However, in this study, none of the volunteers reached protective antibody titer levels, possibly due to the low dose of HBsAg administered (less than 2.2 µg in 3 doses) or the lack of adjuvants.

The efficiency of orally administered plant-based vaccines against chronic HBV infection has also been investigated, based on the ability of the HBcAg of assembly into highly immunogenic capsid-like particles, which were shown to activate the immune system after parenteral [101] or mucosal [96] administration of purified HBcAg. More recently, the efficacy of plant-produced HBcAg as a parenteral-oral vaccine was evaluated, in which HBcAg in *N. benthamiana* extract was injected as a priming dose, followed by boosting with oral administration of lettuce expressing HBcAg, which was shown to induce high titers of serum anti-HBc antibodies (>25,000 mUI/mL), as well as a predominantly Th1 response that is particularly useful in treatment of chronic infections [102].

Altogether, these results suggest that oral administration of HBsAg-expressing plants could function as an effective and low-cost alternative to booster vaccination. However, the immunization protocols need to be further optimized, particularly in regard to the dosage and mucosal adjuvant, before such vaccines could become useful in clinical practice.

### 5.2. Oral Vaccine Candidates against HCV

In contrast to the enormous data accumulated on HBV antigens, only a few attempts have been made to express HCV envelope proteins in plants, most probably, due to the complexity of E1 and E2 folding and assembly. Thus, initial studies have focused on production of immunogenic peptides rather than of entire proteins and do not necessarily involve an oral administration of antigens. Nevertheless, these studies are important for highlighting the potential use and development of HCV antigens as plant produced oral vaccine boosters.

An HCV mimotope generated by fusing the E2 HVR1 epitope to the B subunit of cholera toxin (CTB) was expressed in *N. benthamiana* and further used as a crude extract for intranasal immunization. Interestingly, immunized mice developed antibodies that recognized different HCV virus particles genotypes [103]. Although these antibodies failed to neutralize the HCV infection, the study provided important knowledge regarding the use of adjuvants for mucosal administration of HCV chimeric antigens. Core-derived peptides have also been successfully expressed in tobacco plants, either alone, or fused to HBsAg while preserving their native antigenicity [104]. Madesis et al. successfully expressed a long N-terminal sequence of the Core antigen in chloroplasts of tobacco plants with good antigenicity [104]. Another study showed how the Core protein can be fused to HBsAg and be expressed in tobacco plants using a potato virus X-based vector [105]. One study showed how a crude extract of *N. tabacum* infected with the Cucumber mosaic virus (CMV), expressing an HCV epitope on its surface, can stimulate cultured blood mononuclear cells isolated from chronically HCV infected patients, thus strengthening the hypothesis that plant-derived antigens are potent immunological boosters [106]. The same group reveals that this CMV-based antigen has a good gastric stability and has potential use as an oral vaccine booster [107,108]. More recently, a study has explored the immunogenic properties of canola seed-produced HCV core protein, along with an *E. coli* produced counterpart indicating the interesting property of canola oil-bodies as adjuvants in core immunization strategies [109]. 

It is worth noting that while strategies to produce HCV proteins in plants have advanced a lot, the immunogenicity and virus neutralization to confirm correct structural and functional properties of the antigen and the efficacy of the immune response are mostly lacking. Our group has shown for the first time that the full length E2 can be expressed in plant-based platforms either as a E1E2 dimer or alone, while retaining the molecular and functional properties of the native antigen [18,21]. Importantly, plant produced HCV proteins induced a strong humoral immune response in vaccinated mice when used as a boost following parenteral immunization with the mammalian cell-derived antigen. This highlights the potential use of plant-expressed HCV antigens and reveals the feasibility of using these antigens to boost an initiated immune response. 

We believe that integrating recent design strategies, such as stabilizing key E2 immunodominant epitopes, improving E1E2 expression, and using engineered plants to allow for mammalian-like post-translational modifications such as humanized N-glycosylation [110], will smooth the way to the development of a plant HCV vaccine. Nevertheless, further work should fill the gap between finding the perfect HCV immunogen and expressing it at sufficient levels in plants while retaining its native properties.

## 6. Conclusions

While the proof of concept that an immune response induced by oral vaccines has all the required properties to neutralize viral infections is well documented, making this immunity efficient is still problematic. Public concerns regarding the use of genetically modified plants and environmental risk assessments should also be considered for the economical evaluation of these vaccines’ production on a large scale [111].

Nevertheless, progress has been made during the last decade and extensive research regarding the use of delivery systems and adjuvants has improved the effectiveness of oral vaccination. Previous obstacles, such as the harsh gastro-intestinal environment and oral tolerance, now appear surmountable. A continuous exchange of information between preclinical research and clinical vaccine development is needed to secure improved formulations and adjuvants for the next generation of effective mucosal vaccines. Most importantly, optimization of administration protocols, even combining parenteral with oral delivery, which can not only offer systemic and local protection at the site of infection but can also decrease the number of injections, should contribute to a better vaccination outcome at reduced costs.

## References

[1] Hardt K., Bonanni P., King S., Santos J.I., El-Hodhod M., Zimet G.D., Preiss S. (2016). Vaccine strategies: Optimising outcomes. Vaccine.

[2] McGhee J.R., Fujihashi K. (2012). Inside the mucosal immune system. PLoS Biol..

[3] Lycke N. (2012). Recent progress in mucosal vaccine development: Potential and limitations. Nat. Rev. Immunol..

[4] World Health Organization Hepatitis B Factsheet. http://www.who.int/news-room/fact-sheets/detail/hepatitis-b.

[5] Joung Y.H., Park S.H., Moon K.B., Jeon J.H., Cho H.S., Kim H.S. (2016). The Last Ten Years of Advancements in Plant-Derived Recombinant Vaccines against Hepatitis B. Int. J. Mol. Sci..

[6] Holmgren J., Czerkinsky C. (2005). Mucosal immunity and vaccines. Nat. Med..

[7] Randall T.D., Mebius R.E. (2014). The development and function of mucosal lymphoid tissues: A balancing act with micro-organisms. Mucosal Immunol..

[8] Ogra P.L., Faden H., Welliver R.C. (2001). Vaccination strategies for mucosal immune responses. Clin. Microbiol. Rev..

[9] Macpherson A.J., Uhr T. (2004). Induction of protective IgA by intestinal dendritic cells carrying commensal bacteria. Science.

[10] Dillon A., Lo D.D. (2019). M Cells: Intelligent Engineering of Mucosal Immune Surveillance. Front. Immunol..

[11] Kunisawa J., Kurashima Y., Kiyono H. (2012). Gut-associated lymphoid tissues for the development of oral vaccines. Adv. Drug Deliv. Rev..

[12] Cauley L.S., Lefrançois L. (2013). Guarding the perimeter: Protection of the mucosa by tissue-resident memory T cells. Mucosal Immunol..

[13] Van Ginkel F.W., Nguyen H.H., McGhee J.R. (2000). Vaccines for mucosal immunity to combat emerging infectious diseases. Emerg. Infect. Dis..

[14] Sun M., He C., Cong Y., Liu Z. (2015). Regulatory immune cells in regulation of intestinal inflammatory response to microbiota. Mucosal Immunol..

[15] Vela Ramirez J.E., Sharpe L.A., Peppas N.A. (2017). Current state and challenges in developing oral vaccines. Adv. Drug Deliv. Rev..

[16] Kang S.H., Hong S.J., Lee Y.K., Cho S. (2018). Oral Vaccine Delivery for Intestinal Immunity-Biological Basis, Barriers, Delivery System, and M Cell Targeting. Polymers.

[17] He W., Baysal C., Lobato Gómez M., Huang X., Alvarez D., Zhu C., Armario-Najera V., Blanco Perera A., Cerda Bennaser P., Saba-Mayoral A. (2021). Contributions of the international plant science community to the fight against infectious diseases in humans-part 2: Affordable drugs in edible plants for endemic and re-emerging diseases. Plant Biotechnol. J..

[18] Clarke J.L., Paruch L., Dobrica M.O., Caras I., Tucureanu C., Onu A., Ciulean S., Stavaru C., Eerde A., Wang Y. (2017). Lettuce-produced hepatitis C virus E1E2 heterodimer triggers immune responses in mice and antibody production after oral vaccination. Plant Biotechnol. J..

[19] Marsian J., Lomonossoff G.P. (2016). Molecular pharming—VLPs made in plants. Curr. Opin. Biotechnol..

[20] Van Eerde A., Gottschamel J., Bock R., Hansen K.E.A., Munang’andu H.M., Daniell H., Liu Clarke J. (2019). Production of tetravalent dengue virus envelope protein domain III based antigens in lettuce chloroplasts and immunologic analysis for future oral vaccine development. Plant Biotechnol. J..

[21] Dobrica M.O., van Eerde A., Tucureanu C., Onu A., Paruch L., Caras I., Vlase E., Steen H., Haugslien S., Alonzi D. (2021). Hepatitis C virus E2 envelope glycoprotein produced in *Nicotiana benthamiana* triggers humoral response with virus-neutralizing activity in vaccinated mice. Plant Biotechnol. J..

[22] Sander V.A., Corigliano M.G., Clemente M. (2019). Promising Plant-Derived Adjuvants in the Development of Coccidial Vaccines. Front. Vet. Sci..

[23] Dobrica M.O., Lazar C., Paruch L., Skomedal H., Steen H., Haugslien S., Tucureanu C., Caras I., Onu A., Ciulean S. (2017). A novel chimeric Hepatitis B virus S/preS1 antigen produced in mammalian and plant cells elicits stronger humoral and cellular immune response than the standard vaccine-constituent, S protein. Antivir. Res..

[24] Fausther-Bovendo H., Kobinger G. (2021). Plant-made vaccines and therapeutics. Science.

[25] Daniell H., Jin S., Zhu X.G., Gitzendanner M.A., Soltis D.E., Soltis P.S. (2021). Green giant-a tiny chloroplast genome with mighty power to produce high-value proteins: History and phylogeny. Plant Biotechnol. J..

[26] Su H., Yakovlev I.A., van Eerde A., Su J., Clarke J.L. (2021). Plant-Produced Vaccines: Future Applications in Aquaculture. Front. Plant Sci..

[27] Daniell H., Lin C.-S., Yu M., Chang W.-J. (2016). Chloroplast genomes: Diversity, evolution, and applications in genetic engineering. Genome Biol..

[28] Daniell H., Kulis M., Herzog R.W. (2019). Plant cell-made protein antigens for induction of Oral tolerance. Biotechnol. Adv..

[29] Shahid N., Daniell H. (2016). Plant-based oral vaccines against zoonotic and non-zoonotic diseases. Plant Biotechnol. J..

[30] Czyż M., Dembczyński R., Marecik R., Wojas-Turek J., Milczarek M., Pajtasz-Piasecka E., Wietrzyk J., Pniewski T. (2014). Freeze-drying of plant tissue containing HBV surface antigen for the oral vaccine against hepatitis B. BioMed Res. Int..

[31] Hayden C.A., Smith E.M., Turner D.D., Keener T.K., Wong J.C., Walker J.H., Tizard I.R., Jimenez-Flores R., Howard J.A. (2014). Supercritical fluid extraction provides an enhancement to the immune response for orally-delivered hepatitis B surface antigen. Vaccine.

[32] Lv Z., Jiang R., Chen J., Chen W. (2020). Nanoparticle-mediated gene transformation strategies for plant genetic engineering. Plant J..

[33] Dobrica M.O., Lazar C., Paruch L., van Eerde A., Clarke J.L., Tucureanu C., Caras I., Ciulean S., Onu A., Tofan V. (2018). Oral administration of a chimeric Hepatitis B Virus S/preS1 antigen produced in lettuce triggers infection neutralizing antibodies in mice. Vaccine.

[34] Stanaway J.D., Flaxman A.D., Naghavi M., Fitzmaurice C., Vos T., Abubakar I., Abu-Raddad L.J., Assadi R., Bhala N., Cowie B. (2016). The global burden of viral hepatitis from 1990 to 2013: Findings from the Global Burden of Disease Study 2013. Lancet.

[35] World Health Organization (2017). Global Hepatitis Report, 2017.

[36] Dane D.S., Cameron C.H., Briggs M. (1970). Virus-like particles in serum of patients with Australia-antigen-associated hepatitis. Lancet.

[37] Robinson W.S., Clayton D.A., Greenman R.L. (1974). DNA of a human hepatitis B virus candidate. J. Virol..

[38] Summers J., O’Connell A., Millman I. (1975). Genome of hepatitis B virus: Restriction enzyme cleavage and structure of DNA extracted from Dane particles. Proc. Natl. Acad. Sci. USA.

[39] Karayiannis P. (2017). Hepatitis B virus: Virology, molecular biology, life cycle and intrahepatic spread. Hepatol. Int..

[40] Seeger C., Mason W.S. (2000). Hepatitis B virus biology. Microbiol. Mol. Biol. Rev..

[41] Neurath A.R., Kent S.B., Strick N., Parker K. (1986). Identification and chemical synthesis of a host cell receptor binding site on hepatitis B virus. Cell.

[42] Gripon P., Cannie I., Urban S. (2005). Efficient inhibition of hepatitis B virus infection by acylated peptides derived from the large viral surface protein. J. Virol..

[43] Yato K., Onodera T., Matsuda M., Moriyama S., Fujimoto A., Watashi K., Aizaki H., Tanaka T., Moriishi K., Nishitsuji H. (2020). Identification of Two Critical Neutralizing Epitopes in the Receptor Binding Domain of Hepatitis B Virus preS1. J. Virol..

[44] Krugman S. (1982). The newly licensed hepatitis B vaccine. Characteristics and indications for use. Jama.

[45] McAleer W.J., Buynak E.B., Maigetter R.Z., Wampler D.E., Miller W.J., Hilleman M.R. (1984). Human hepatitis B vaccine from recombinant yeast. Nature.

[46] Kim W.R. (2009). Epidemiology of hepatitis B in the United States. Hepatology.

[47] Rubin L.G., Levin M.J., Ljungman P., Davies E.G., Avery R., Tomblyn M., Bousvaros A., Dhanireddy S., Sung L., Keyserling H. (2014). 2013 IDSA clinical practice guideline for vaccination of the immunocompromised host. Clin. Infect. Dis..

[48] Sahana H.V., Sarala N., Prasad S.R. (2017). Decrease in Anti-HBs Antibodies over Time in Medical Students and Healthcare Workers after Hepatitis B Vaccination. BioMed Res. Int..

[49] Shouval D., Roggendorf H., Roggendorf M. (2015). Enhanced immune response to hepatitis B vaccination through immunization with a Pre-S1/Pre-S2/S vaccine. Med. Microbiol. Immunol..

[50] Yum J.S., Ahn B.C., Jo H.J., Kim D.Y., Kim K.H., Kim H.S., Sung Y.C., Yoon J., Morrey J., Moon H.M. (2012). Use of pre-S protein-containing hepatitis B virus surface antigens and a powerful adjuvant to develop an immune therapy for chronic hepatitis B virus infection. Clin. Vaccine Immunol..

[51] Shapira M.Y., Zeira E., Adler R., Shouval D. (2001). Rapid seroprotection against hepatitis B following the first dose of a Pre-S1/Pre-S2/S vaccine. J. Hepatol..

[52] Qian B., Shen H., Liang W., Guo X., Zhang C., Wang Y., Li G., Wu A., Cao K., Zhang D. (2008). Immunogenicity of recombinant hepatitis B virus surface antigen fused with preS1 epitopes expressed in rice seeds. Transgenic Res..

[53] Rendi-Wagner P., Shouval D., Genton B., Lurie Y., Rümke H., Boland G., Cerny A., Heim M., Bach D., Schroeder M. (2006). Comparative immunogenicity of a PreS/S hepatitis B vaccine in non- and low responders to conventional vaccine. Vaccine.

[54] Abramowicz M., Zuccotti G., Pflomm J.M. (2018). A two-dose hepatitis B vaccine for adults (Heplisav-B). Med. Lett. Drugs Ther..

[55] Champion C.R. (2021). Heplisav-B: A Hepatitis B Vaccine With a Novel Adjuvant. Ann. Pharmacother..

[56] Choo Q.L., Kuo G., Weiner A.J., Overby L.R., Bradley D.W., Houghton M. (1989). Isolation of a cDNA clone derived from a blood-borne non-A, non-B viral hepatitis genome. Science.

[57] Chevaliez S., Pawlotsky J.M., Tan S.L. (2006). HCV Genome and Life Cycle. Hepatitis C Viruses: Genomes and Molecular Biology.

[58] Agnello V., Abel G., Elfahal M., Knight G.B., Zhang Q.X. (1999). Hepatitis C virus and other flaviviridae viruses enter cells via low density lipoprotein receptor. Proc. Natl. Acad. Sci. USA.

[59] Monazahian M., Böhme I., Bonk S., Koch A., Scholz C., Grethe S., Thomssen R. (1999). Low density lipoprotein receptor as a candidate receptor for hepatitis C virus. J. Med. Virol..

[60] Blanchard E., Belouzard S., Goueslain L., Wakita T., Dubuisson J., Wychowski C., Rouillé Y. (2006). Hepatitis C virus entry depends on clathrin-mediated endocytosis. J. Virol..

[61] Farquhar M.J., Hu K., Harris H.J., Davis C., Brimacombe C.L., Fletcher S.J., Baumert T.F., Rappoport J.Z., Balfe P., McKeating J.A. (2012). Hepatitis C virus induces CD81 and claudin-1 endocytosis. J. Virol..

[62] Keck Z.Y., Xia J., Wang Y., Wang W., Krey T., Prentoe J., Carlsen T., Li A.Y., Patel A.H., Lemon S.M. (2012). Human monoclonal antibodies to a novel cluster of conformational epitopes on HCV E2 with resistance to neutralization escape in a genotype 2a isolate. PLoS Pathog..

[63] Tsukiyama-Kohara K., Kohara M. (2018). Hepatitis C Virus: Viral Quasispecies and Genotypes. Int. J. Mol. Sci..

[64] Martell M., Esteban J.I., Quer J., Genescà J., Weiner A., Esteban R., Guardia J., Gómez J. (1992). Hepatitis C virus (HCV) circulates as a population of different but closely related genomes: Quasispecies nature of HCV genome distribution. J. Virol..

[65] Farci P., Bukh J., Purcell R.H. (1997). The quasispecies of hepatitis C virus and the host immune response. Springer Semin. Immunopathol..

[66] Liu L., Fisher B.E., Dowd K.A., Astemborski J., Cox A.L., Ray S.C. (2010). Acceleration of hepatitis C virus envelope evolution in humans is consistent with progressive humoral immune selection during the transition from acute to chronic infection. J. Virol..

[67] Micallef J.M., Kaldor J.M., Dore G.J. (2006). Spontaneous viral clearance following acute hepatitis C infection: A systematic review of longitudinal studies. J. Viral Hepat..

[68] Osburn W.O., Fisher B.E., Dowd K.A., Urban G., Liu L., Ray S.C., Thomas D.L., Cox A.L. (2010). Spontaneous control of primary hepatitis C virus infection and immunity against persistent reinfection. Gastroenterology.

[69] Sacks-Davis R., Grebely J., Dore G.J., Osburn W., Cox A.L., Rice T.M., Spelman T., Bruneau J., Prins M., Kim A.Y. (2015). Hepatitis C Virus Reinfection and Spontaneous Clearance of Reinfection—The InC3 Study. J. Infect. Dis..

[70] Holz L., Rehermann B. (2015). T cell responses in hepatitis C virus infection: Historical overview and goals for future research. Antivir. Res..

[71] Bankwitz D., Steinmann E., Bitzegeio J., Ciesek S., Friesland M., Herrmann E., Zeisel M.B., Baumert T.F., Keck Z.Y., Foung S.K. (2010). Hepatitis C virus hypervariable region 1 modulates receptor interactions, conceals the CD81 binding site, and protects conserved neutralizing epitopes. J. Virol..

[72] Bankwitz D., Vieyres G., Hueging K., Bitzegeio J., Doepke M., Chhatwal P., Haid S., Catanese M.T., Zeisel M.B., Nicosia A. (2014). Role of hypervariable region 1 for the interplay of hepatitis C virus with entry factors and lipoproteins. J. Virol..

[73] Lavie M., Hanoulle X., Dubuisson J. (2018). Glycan Shielding and Modulation of Hepatitis C Virus Neutralizing Antibodies. Front. Immunol..

[74] Owsianka A., Tarr A.W., Juttla V.S., Lavillette D., Bartosch B., Cosset F.L., Ball J.K., Patel A.H. (2005). Monoclonal antibody AP33 defines a broadly neutralizing epitope on the hepatitis C virus E2 envelope glycoprotein. J. Virol..

[75] Pantua H., Diao J., Ultsch M., Hazen M., Mathieu M., McCutcheon K., Takeda K., Date S., Cheung T.K., Phung Q. (2013). Glycan shifting on hepatitis C virus (HCV) E2 glycoprotein is a mechanism for escape from broadly neutralizing antibodies. J. Mol. Biol..

[76] Keck Z.Y., Pierce B.G., Lau P., Lu J., Wang Y., Underwood A., Bull R.A., Prentoe J., Velázquez-Moctezuma R., Walker M.R. (2019). Broadly neutralizing antibodies from an individual that naturally cleared multiple hepatitis C virus infections uncover molecular determinants for E2 targeting and vaccine design. PLoS Pathog..

[77] Choo Q.L., Kuo G., Ralston R., Weiner A., Chien D., Van Nest G., Han J., Berger K., Thudium K., Kuo C. (1994). Vaccination of chimpanzees against infection by the hepatitis C virus. Proc. Natl. Acad. Sci. USA.

[78] Frey S.E., Houghton M., Coates S., Abrignani S., Chien D., Rosa D., Pileri P., Ray R., Di Bisceglie A.M., Rinella P. (2010). Safety and immunogenicity of HCV E1E2 vaccine adjuvanted with MF59 administered to healthy adults. Vaccine.

[79] Guest J.D., Wang R., Elkholy K.H., Chagas A., Chao K.L., Cleveland T.E., Kim Y.C., Keck Z.Y., Marin A., Yunus A.S. (2021). Design of a native-like secreted form of the hepatitis C virus E1E2 heterodimer. Proc. Natl. Acad. Sci. USA.

[80] Mason H.S., Lam D.M., Arntzen C.J. (1992). Expression of hepatitis B surface antigen in transgenic plants. Proc. Natl. Acad. Sci. USA.

[81] Thanavala Y., Yang Y.F., Lyons P., Mason H.S., Arntzen C. (1995). Immunogenicity of transgenic plant-derived hepatitis B surface antigen. Proc. Natl. Acad. Sci. USA.

[82] Kapusta J., Modelska A., Figlerowicz M., Pniewski T., Letellier M., Lisowa O., Yusibov V., Koprowski H., Plucienniczak A., Legocki A.B. (1999). A plant-derived edible vaccine against hepatitis B virus. FASEB J..

[83] Richter L.J., Thanavala Y., Arntzen C.J., Mason H.S. (2000). Production of hepatitis B surface antigen in transgenic plants for oral immunization. Nat. Biotechnol..

[84] Joung Y.H., Youm J.W., Jeon J.H., Lee B.C., Ryu C.J., Hong H.J., Kim H.C., Joung H., Kim H.S. (2004). Expression of the hepatitis B surface S and preS2 antigens in tubers of Solanum tuberosum. Plant Cell Rep..

[85] Youm J.W., Won Y.S., Jeon J.H., Ryu C.J., Choi Y.K., Kim H.C., Kim B.D., Joung H., Kim H.S. (2007). Oral immunogenicity of potato-derived HBsAg middle protein in BALB/c mice. Vaccine.

[86] Rukavtsova E.B., Rudenko N.V., Puchko E.N., Zakharchenko N.S., Buryanov Y.I. (2015). Study of the immunogenicity of hepatitis B surface antigen synthesized in transgenic potato plants with increased biosafety. J. Biotechnol..

[87] Kong Q., Richter L., Yang Y.F., Arntzen C.J., Mason H.S., Thanavala Y. (2001). Oral immunization with hepatitis B surface antigen expressed in transgenic plants. Proc. Natl. Acad. Sci. USA.

[88] Gao Y., Ma Y., Li M., Cheng T., Li S.W., Zhang J., Xia N.S. (2003). Oral immunization of animals with transgenic cherry tomatillo expressing HBsAg. World J. Gastroenterol..

[89] Salyaev R.K., Stolbikov A.S., Rekoslavskaya N.I., Shchelkunov S.N., Pozdnyakov S.G., Chepinoga A.V., Hammond R.V. (2010). Obtaining tomato plants transgenic for the preS2-S-HDEL gene, which synthesize the major hepatitis B surface antigen. Dokl. Biochem. Biophys..

[90] Hayden C.A., Streatfield S.J., Lamphear B.J., Fake G.M., Keener T.K., Walker J.H., Clements J.D., Turner D.D., Tizard I.R., Howard J.A. (2012). Bioencapsulation of the hepatitis B surface antigen and its use as an effective oral immunogen. Vaccine.

[91] Hayden C.A., Fischer M.E., Andrews B.L., Chilton H.C., Turner D.D., Walker J.H., Tizard I.R., Howard J.A. (2015). Oral delivery of wafers made from HBsAg-expressing maize germ induces long-term immunological systemic and mucosal responses. Vaccine.

[92] Kapusta J., Modelska A., Pniewski T., Figlerowicz M., Jankowski K., Lisowa O., Plucienniczak A., Koprowski H., Legocki A.B. (2001). Oral immunization of human with transgenic lettuce expressing hepatitis B surface antigen. Adv. Exp. Med. Biol..

[93] Pniewski T., Kapusta J., Bociąg P., Wojciechowicz J., Kostrzak A., Gdula M., Fedorowicz-Strońska O., Wójcik P., Otta H., Samardakiewicz S. (2011). Low-dose oral immunization with lyophilized tissue of herbicide-resistant lettuce expressing hepatitis B surface antigen for prototype plant-derived vaccine tablet formulation. J. Appl. Genet..

[94] Pniewski T., Milczarek M., Wojas-Turek J., Pajtasz-Piasecka E., Wietrzyk J., Czyż M. (2018). Plant lyophilisate carrying S-HBsAg as an oral booster vaccine against HBV. Vaccine.

[95] Salyaev R.K., Rekoslavskaya N.I., Stolbikov A.S., Tret’yakova A.V. (2012). Candidate mucosal vaccine against hepatitis B based on tomatoes transgenic for the preS2-S gene. Dokl. Biochem. Biophys..

[96] Huang Z., Santi L., LePore K., Kilbourne J., Arntzen C.J., Mason H.S. (2006). Rapid, high-level production of hepatitis B core antigen in plant leaf and its immunogenicity in mice. Vaccine.

[97] Thanavala Y., Mahoney M., Pal S., Scott A., Richter L., Natarajan N., Goodwin P., Arntzen C.J., Mason H.S. (2005). Immunogenicity in humans of an edible vaccine for hepatitis B. Proc. Natl. Acad. Sci. USA.

[98] Salyaev K., Rekoslavskaya N.I., Stolbikov A.S., Hammond R.W., Shchelkunov S.N. (2007). Synthesis of hepatitis B virus surface antigen in tomato plants transgenic for the preS2-S gene. Dokl. Biochem. Biophys..

[99] Cui X.X., Yang X., Wang H.J., Rong X.Y., Jing S., Xie Y.H., Huang D.F., Zhao C. (2017). Luteolin-7-O-Glucoside Present in Lettuce Extracts Inhibits Hepatitis B Surface Antigen Production and Viral Replication by Human Hepatoma Cells in Vitro. Front. Microbiol..

[100] Pniewski T., Kapusta J., Bociąg P., Kostrzak A., Fedorowicz-Strońska O., Czyż M., Gdula M., Krajewski P., Wolko B., Płucienniczak A. (2012). Plant expression, lyophilisation and storage of HBV medium and large surface antigens for a prototype oral vaccine formulation. Plant Cell Rep..

[101] Buchmann P., Dembek C., Kuklick L., Jäger C., Tedjokusumo R., von Freyend M.J., Drebber U., Janowicz Z., Melber K., Protzer U. (2013). A novel therapeutic hepatitis B vaccine induces cellular and humoral immune responses and breaks tolerance in hepatitis B virus (HBV) transgenic mice. Vaccine.

[102] Pyrski M., Mieloch A.A., Plewiński A., Basińska-Barczak A., Gryciuk A., Bociąg P., Murias M., Rybka J.D., Pniewski T. (2019). Parenteral-Oral Immunization with Plant-Derived HBcAg as a Potential Therapeutic Vaccine against Chronic Hepatitis B. Vaccines.

[103] Nemchinov L.G., Liang T.J., Rifaat M.M., Mazyad H.M., Hadidi A., Keith J.M. (2000). Development of a plant-derived subunit vaccine candidate against hepatitis C virus. Arch. Virol..

[104] Madesis P., Osathanunkul M., Georgopoulou U., Gisby M.F., Mudd E.A., Nianiou I., Tsitoura P., Mavromara P., Tsaftaris A., Day A. (2010). A hepatitis C virus core polypeptide expressed in chloroplasts detects anti-core antibodies in infected human sera. J. Biotechnol..

[105] Mohammadzadeh S., Roohvand F., Memarnejadian A., Jafari A., Ajdary S., Salmanian A.H., Ehsani P. (2016). Co-expression of hepatitis C virus polytope-HBsAg and p19-silencing suppressor protein in tobacco leaves. Pharm. Biol..

[106] Denis J., Majeau N., Acosta-Ramirez E., Savard C., Bedard M.C., Simard S., Lecours K., Bolduc M., Pare C., Willems B. (2007). Immunogenicity of papaya mosaic virus-like particles fused to a hepatitis C virus epitope: Evidence for the critical function of multimerization. Virology.

[107] Nuzzaci M., Piazzolla G., Vitti A., Lapelosa M., Tortorella C., Stella I., Natilla A., Antonaci S., Piazzolla P. (2007). Cucumber mosaic virus as a presentation system for a double hepatitis C virus-derived epitope. Arch. Virol..

[108] Nuzzaci M., Vitti A., Condelli V., Lanorte M.T., Tortorella C., Boscia D., Piazzolla P., Piazzolla G. (2010). In vitro stability of Cucumber mosaic virus nanoparticles carrying a Hepatitis C virus-derived epitope under simulated gastrointestinal conditions and in vivo efficacy of an edible vaccine. J. Virol. Methods.

[109] Mohammadzadeh S., Roohvand F., Ehsani P., Salmanian A.H., Ajdary S. (2020). *Canola* oilseed- and *Escherichia coli*- derived hepatitis C virus (HCV) core proteins adjuvanted with oil bodies, induced robust Th1-oriented immune responses in immunized mice. APMIS.

[110] Kim H.S., Jeon J.H., Lee K.J., Ko K. (2014). N-glycosylation modification of plant-derived virus-like particles: An application in vaccines. BioMed Res. Int..

[111] Venkataraman S., Hefferon K., Makhzoum A., Abouhaidar M. (2021). Combating Human Viral Diseases: Will Plant-Based Vaccines Be the Answer?. Vaccines.

